# Bacterial and fungal communities of traditional fermented Chinese soybean paste (Doujiang) and their properties

**DOI:** 10.1002/fsn3.2505

**Published:** 2021-08-31

**Authors:** Fei Ren, Dong‐Hui Yan, Yuchun Liu, Chao Wang, Chao Guo

**Affiliations:** ^1^ Institute of Cereal & Oil Science and Technology Academy of National Food and Strategic Reserves Administration Beijing China; ^2^ The Key Laboratory of Forest Protection affiliated to State Forestry Administration of China Institute of Forest Ecology Environment and Protection Chinese Academy of Forestry Beijing China

**Keywords:** 16S and ITS rDNA amplicon sequencing, fermented soybean paste, microbial community diversity, protease‐producing microbes

## Abstract

Soybean paste (Doujiang) is one of the traditional fermented foods from China, fermented by various microorganisms. However, the microflora of Doujiang keeps little known. In this study, the microbial communities of seven kinds of representative Doujiang samples were investigated by both culture‐independent and culture‐dependent methods. We found that core OTUs among seven Doujiang samples were mainly from *Bacillus*, *Pseudomonas*, *Candida,* and *Aspergillus* according to Illumina sequencing. Every type of Doujiang sample harbored a different composition of microbial community. Doujiang LSJ and LBJ had the highest bacterial and fungal richness and diversity, respectively. The structure of microbial community was remarkably correlated with Doujiang properties—pH, and the content of total protein, soluble protein, amino acid, and total sugar (*p* < .05). *Bacillus* spp. were most frequently isolated bacterial species. Fungi of *Monascus*, *Candida,* and *Aspergillus* were also isolated. Eleven microbial strains showed high protease activities to degrade corn proteins, which can form obvious transparent hydrolytic circles in corn gluten meal medium plates. Therefore, microbial communities were supposed to tightly connect to Doujiang type and properties. It is possible to apply potential protein‐degrading microbial strains to corn byproducts for protein production in the future study.

## Graphical abstract

Every investigated soybean paste harbored a distinctive microbial community. The structure of microbial community was remarkably correlated with pH, content of total protein, soluble protein, amino acid, and total sugar.

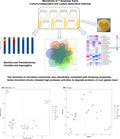

## 1 INTRODUCTION

The soybean paste (Doujiang) is one of the favorite traditional fermented foods originated from China with a long history and is very popular in China as well as Asian countries (He et al., 2017). Doujiang is mainly made from soybean and fermented by many kinds of microorganisms, containing a lot of active substances good to human health (He et al., 2017; Zhang et al., 2018). The traditional fermentation process is a time‐consuming way to achieve desirable flavor through solidarization and liquid fermentation, with the treated soybean materials open to the air utilizing natural microorganisms (Zhang et al., 2018; Zhao et al., 2009). Doujiang generates a complicated mix of microbial assemblage including fungi and bacteria during the production (Gao et al., 2011; Wu et al., 2013). Microbial fermentation could increase shelf life as well as confer beneficial properties for food by flavor, texture, and nutrition (Gao et al., 2011; He et al., 2017; Ma et al., 2019). Thus, microbial fermentation is a productive and economical way for food preservation and processing (He et al., 2017; Nahidul‐Islam et al., 2018).

Microorganisms are the key factor of fermented foods, playing a vital role in the final quality and properties (He et al., 2017). Complex biochemical conversions happen during the fermentation due to the metabolic activities of microbes (Ge et al., 2012; He et al., 2017; Wu et al., 2013). To investigate these microorganisms, culture‐dependent method was a widely and conventional isolation technique (Carvalho et al., 2020; Nahidul‐Islam et al., 2018; Zhu et al., 2019). Meanwhile, with the development of biomolecular technology, PCR‐DGGE technology was adopted in microbial community during fermentation (Gao et al., 2011; Zhao et al., 2009; Zhu et al., 2019). Nowadays, culture‐independent approaches especially high‐throughput sequencing can provide people with a new and comprehensive view into food fermentation systems (Kim et al., 2009; Lee et al., 2019; Rizo et al., 2018).

For their important roles on food quality and flavor determination, the microbes in traditional fermented foods should be broadly investigated and identified (Gao et al., 2011; Ge et al., 2012; Wu et al., 2013; Zhang et al., 2018; Zhao et al., 2009). Determining the complicated microbes of fermented foods and then understanding their specific functions on the food quality is a continuous topic in food microbial study (He et al., 2017). However, information on microflora of these various Doujiang is still very limited (Gao et al., 2011; Ge et al., 2012; Wu et al., 2013; Zhang et al., 2018). We speculate the microbial communities among our sampled Doujiang types should be different. What's more, considering the special flavor and quality of the Doujiang, the structure of microbial community might also correlate with the Doujiang properties. In the study, the composition differences on the microflora of Doujiang samples were investigated. Correlations between the microbial community and Doujiang properties were also studied, and the potential application of isolated strains on corn byproducts was expected.

## 2 MATERIALS AND METHODS

### 2.1 Samples processing

Seven kinds of representative Chinese traditional fermented Doujiang (JDHJ, LBJ, XQ, LSJ, HT, SDCBJ, and HGDJ) from six different provinces were chosen (Table S1). Each Doujiang had three biological replicates. The samples were purchased from local shops (Table S1). For cultural‐dependent isolation, serial dilutions (10^–1^ to 10^–5^) of Doujiang samples with sterilized double distilled water were plated and inoculated in Petri dishes of Luria‐Bertani agar (LB) and potato dextrose agar (PDA) (Morozumi et al., 2010). Petri dishes were sealed, incubated at 37℃ and 25℃ for bacteria and fungi, respectively, and examined periodically. The pH of samples was measured with a pH meter (Mettler Toledo, Switzerland) by mixing the homogenized sauce suspensions in boiled demineralized water with a ratio of 1:1. Concentrations of total protein (P1) and Soluble protein (P2) were tested with Protein Kit and Soluble Protein Kit (Suzhou Grace Biotechnology Co. Ltd, China). Concentrations of amino acid (AA) and total sugar (TS) were tested with Amino Acid Kit and Total Sugar Kit (Suzhou Comin Biotechnology Co. Ltd, China). The protease activity of strains was screened by corn gluten meal medium and Folinol method (Zhu et al., 2019).

### 2.2 DNA extraction, rDNA amplification, and sequencing analysis

Total DNA of the Doujiang samples was extracted with a benzyl chloride method (Zhu et al., 1993). TIANamp Bacteria DNA Kit (TIANGEN Biotech Co. Ltd., China) was used to extract DNA for bacterial isolation strains, while genomic DNA of fungal cultures was extracted with the CTAB (cetyl trimethyl ammonium bromide) method (Chang et al., 1993). The DNA concentrations were measured with NanoDrop One spectrophotometer (Thermo Fisher Scientific, USA).

The bacterial V3‐V4 region of 16S rRNA gene was amplified with the primers 338F (ACTCCTACGGGAGGCAGCA) and 806R (GGACTACHVGGGTWTCTAAT) (Ren et al., 2020). ITS1F (CTTGGTCATTTAGAGGAAGTAA) and ITS2R (GCTGCGTTCTTCATC GATGC) were used to amplify fungal ITS1 region (Costa et al., 2021; White et al. 1990). Mastercycler gradient (Eppendorf, Germany) was used for PCR performance. The cycling parameters were 95℃ for 2 min, 35 cycles of 95℃ for 30 s, 56℃ for 30 s, and 72℃ for 40 s, and then a final extension at 72℃ for 7 min. QIAquick Gel Extraction Kit (QIAGEN, Germany) was used to purify the PCR products, and Real‐Time PCR was used to be quantified. Then, the products were sequenced with Illumina MiSeq PE300 platform at Beijing Allwegene Technology Ltd. The 16S rDNA was amplified using primer pairs 27F and 1492R for isolated bacterial strains (Nyanzi et al., 2013), while the ITS region was amplified using ITS1 and ITS4 for fungal isolated ones (White et al. 1990).

Qualified reads were separated and trimmed with Illumina Analysis Pipeline Version 2.6. The QIIME 1.8 was used to analyze the dataset (Caporaso et al., 2010). Sequences were clustered into OTUs (operational taxonomic units) at a level of 97% similarity and then generate rarefaction curves and calculate the α‐diversity indexes. The sequences were classified into different taxonomic groups by the RDP Database (Wang et al., 2007). Clustering analyses of PCoA, NMDS, PERMANOVA, and Venn diagram were done by R language based on the OTU information from Doujiang (R core team, 2013). The correlation between microbes and Doujiang property was calculated by Spearman algorithm and the correlation heatmap was also drawn by R (R core team, 2013). Taxa that significantly determine the sample structure were found by LEfSe analysis at different level (LEfSe, 2020). The sequences of isolated strains were analyzed and identified by NCBI blast. High‐throughput sequences were submitted to the SRA (Sequence Read Archive) of the NCBI (National Center for Biotechnology Information) under project accession number PRJNA688022. Sequences of cultured strains were under NCBI no. MW368498‐MW368550 and MW368653‐MW368665.

## 3 RESULTS

### 3.1 Data output and α‐diversity of Doujiang microbiota

A total of 1,092,983 bacterial and 1,417,409 fungal sequences were recovered from the Doujiangs after sequence quality evaluation. The average sequences of Doujiangs were 52,046 ± 1702 (bacteria) and 67.495 ± 1849 (fungi) (mean ± *SD*). The average sequence length was 436 bp for bacteria and 278 bp for fungi.

Sequences were clustered into 681 bacterial OTUs and 1,149 fungal OTUs. The Goods coverage indexes are very high (≥0.996) to all seven Doujiang samples (Table 1). LSJ had the highest bacteria Chao 1 and Shannon index, while the sample LBJ had the highest fungal richness Chao 1 and Shannon index (Table 1). The lowest Chao 1 and Shannon were in JDHJ (bacteria) and SDCBJ (fungi). All the Chao 1 and Shannon indexes of fungi were higher than those of bacterial ones among the samples (Table 1). Rarefaction curve illustrated that the OTUs were saturated in all Doujiang samples (Figure S1).

**TABLE 1:** α‐diversity indexes of microbial communities (mean±*SD*)

Samples	Microbes	Chao1	Goods_coverage	Shannon
JDHJ	Bacteria	126.16 ± 10.33	0.999 ± 0.0002	1.86 ± 0.05
	Fungi	513.77 ± 17.62	0.996 ± 0.0004	5.16 ± 0.17
LBJ	Bacteria	209.41 ± 13.33	0.998 ± 0.0007	2.13 ± 0.04
	Fungi	908.23 ± 17.51	0.996 ± 0.0001	5.84 ± 0.13
XQ	Bacteria	193.04 ± 5.67	0.998 ± 0.0004	2.05 ± 0.08
	Fungi	517.31 ± 8.15	0.998 ± 0.0015	5.23 ± 0.23
LSJ	Bacteria	259.95 ± 15.34	0.997 ± 0.0005	3.45 ± 0.07
	Fungi	656.39 ± 23.15	0.997 ± 0.0009	5.77 ± 0.05
HT	Bacteria	167.85 ± 11.67	0.999 ± 0.0002	2.49 ± 0.12
	Fungi	329.69 ± 46.67	0.997 ± 0.0016	5.13 ± 0.14
SDCBJ	Bacteria	193.76 ± 24.61	0.998 ± 0.0008	1.92 ± 0.08
	Fungi	269.37 ± 40.47	0.998 ± 0.0014	5.05 ± 0.22
HGDJ	Bacteria	168.12 ± 10.67	0.999 ± 0.0004	2.75 ± 0.05
	Fungi	269.37 ± 16.33	0.998 ± 0.0006	5.61 ± 0.26

### 3.2 Microbial community composition among different Doujiang samples

**FIGURE 1:**
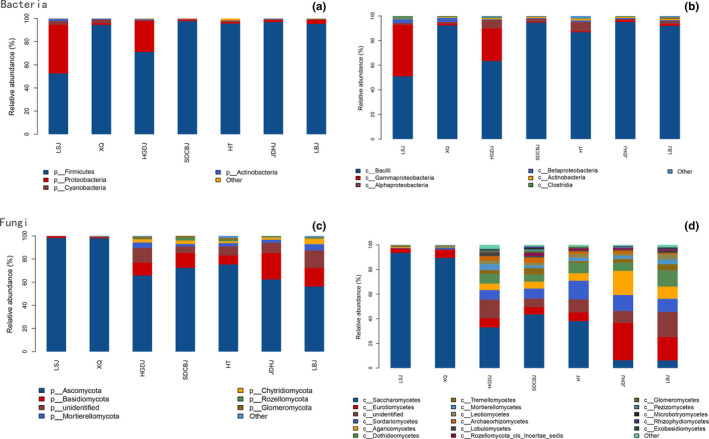
Bacteria abundance in different Doujiang samples: (a) phyla; (b) classes; and fungal abundance in different Doujiang samples: (c) phyla; (d) classes

The sequences belonged to bacteria were classified into 17 phyla. The phyla whose relative abundances more than 1% in samples were in Figure 1A. Firmicutes was most abundant, followed by Proteobacteria. Phyla abundance were different among seven Doujiang samples (Figure 1A). Class Bacilli, Gammaproteobacteria, and Alphaproteobacteria were most abundant (Figure 1B). As to fungi, Phyla Ascomycota was the richest group and then Basidiomycota (Figure 1C). Seventeen classes had a relative abundance exceeding 1% (Figure 1D). Their relative abundance showed great differences among Doujiang samples, including Eurotiomycetes, Sordariomycetes, Agaricomycetes, and Dothideomycetes.

**FIGURE 2:**
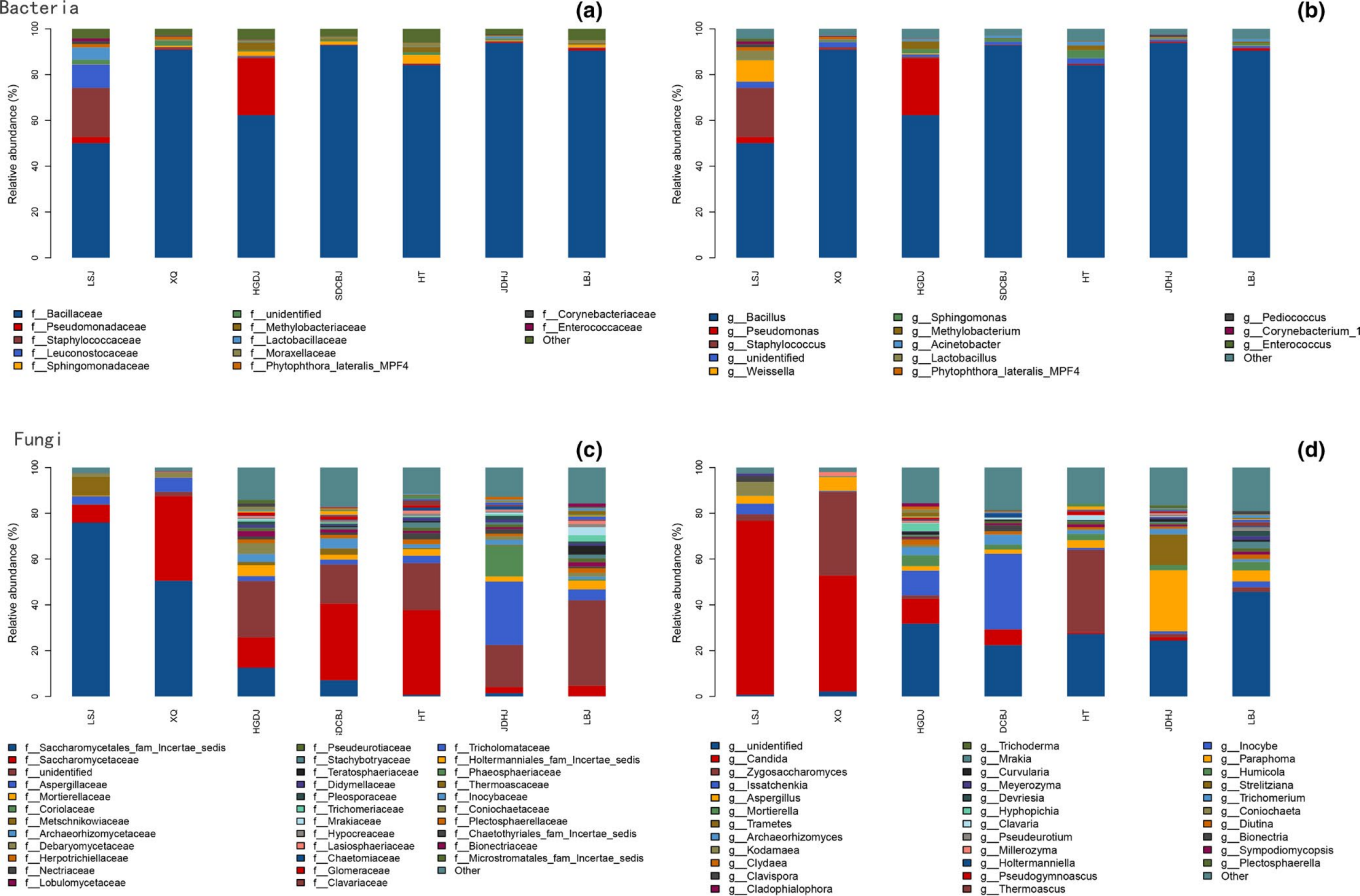
Bacterial abundance in different Doujiang samples: (a) families; (b) genera; fungal abundance in different Doujiang samples: (c) families; (d) genera

At family level, Bacillaceae was most abundant prokaryotic class (Figure 2a). Other families whose relative abundance exceeded 1% are also shown in Figure 2a. Eukaryotic Saccharomycetales_fam_Incertae_sedis group, Saccharomycetaceae, Aspergillaceae were most abundant. For genera, prokaryotic *Bacillus* predominated among Doujiang samples. Eukaryotic *Candida* was most abundant, especially in Doujiang LSJ and XQ; *Aspergillus* was rich among samples, particularly in JDHJ samples.

Among Doujiang samples, significantly different taxa from phylum to genus were found by LEfSe analysis in Figure S2. For example, according to the LDA score, abundance of *Oceanbacillus* in XQ, and abundance of *Bacillus*, *Lactobacillus*, *Enterococcus,* etc., in LSJ were most different biomarkers of bacterial community, while abundance of *Aspergillus* in JDHJ, abundance of genus *Candida* in LSJ, and abundance of *Candida riodocensis* in XQ may contribute most for fungal structure differences (Figure S2).

**FIGURE 3:**
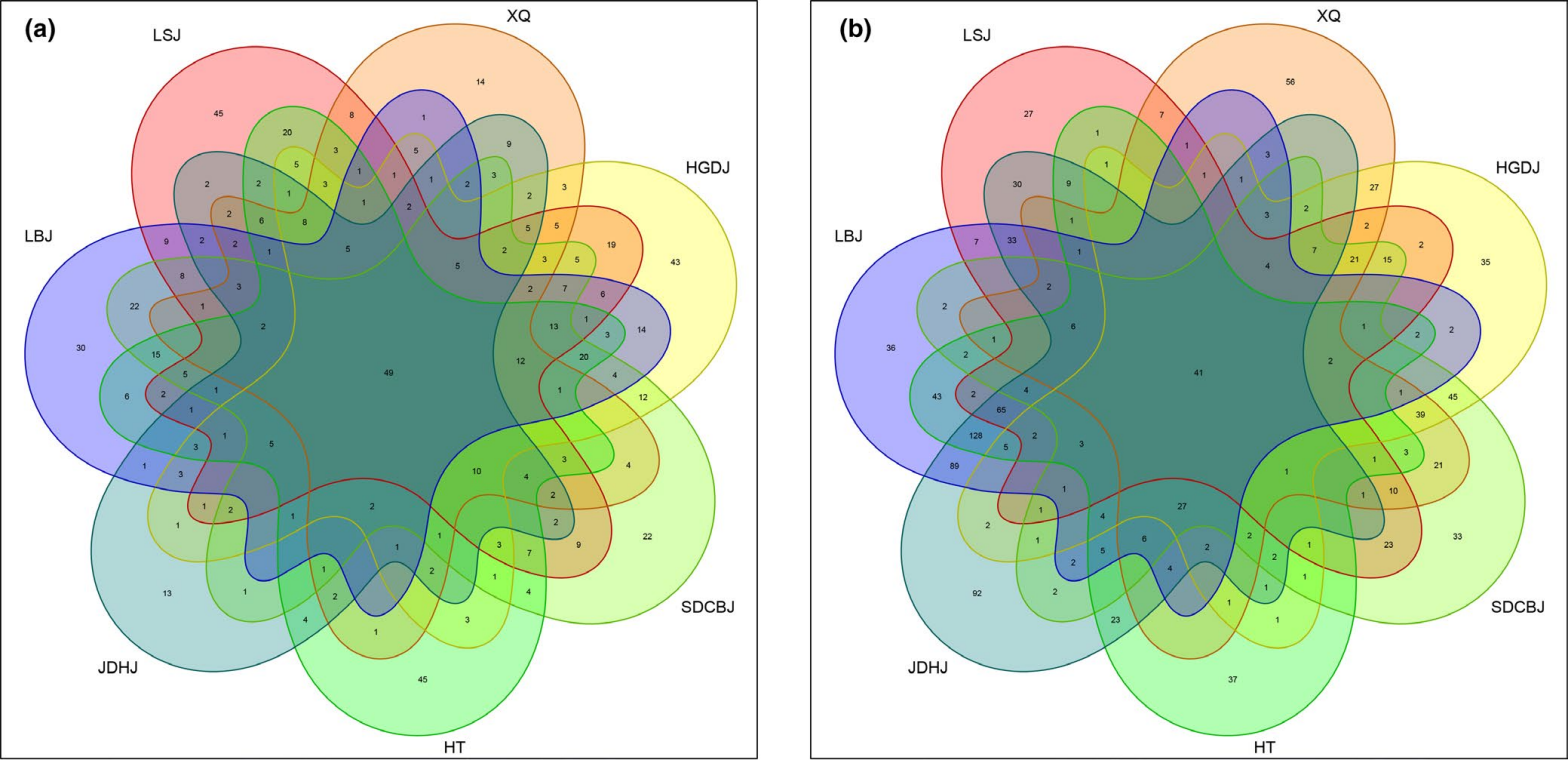
Venn diagram illustrating shared and unique (a) bacterial; (b) fungal OTUs in seven samples

The 49 bacterial and 41 fungal OTUs were found in all seven Doujiang samples. The OTUs unique to a Doujiang sample ranged from 13 bacterial OTUs (JDHJ) and 27 fungal OTUs to 45 bacterial OTUs (HT and LSJ) and 92 fungal OTUs (JDHJ) (Figure 3). Core OTUs across 100% of samples mainly belong to *Bacillus*, *Pseudomonas*, *Candida,* and *Aspergillus*. These core OTUs might play active roles in the fermentation process in the Doujiang samples.

### 3.3 Relationship between structure of microbial community, sample types, and Doujiang properties

**FIGURE 4:**
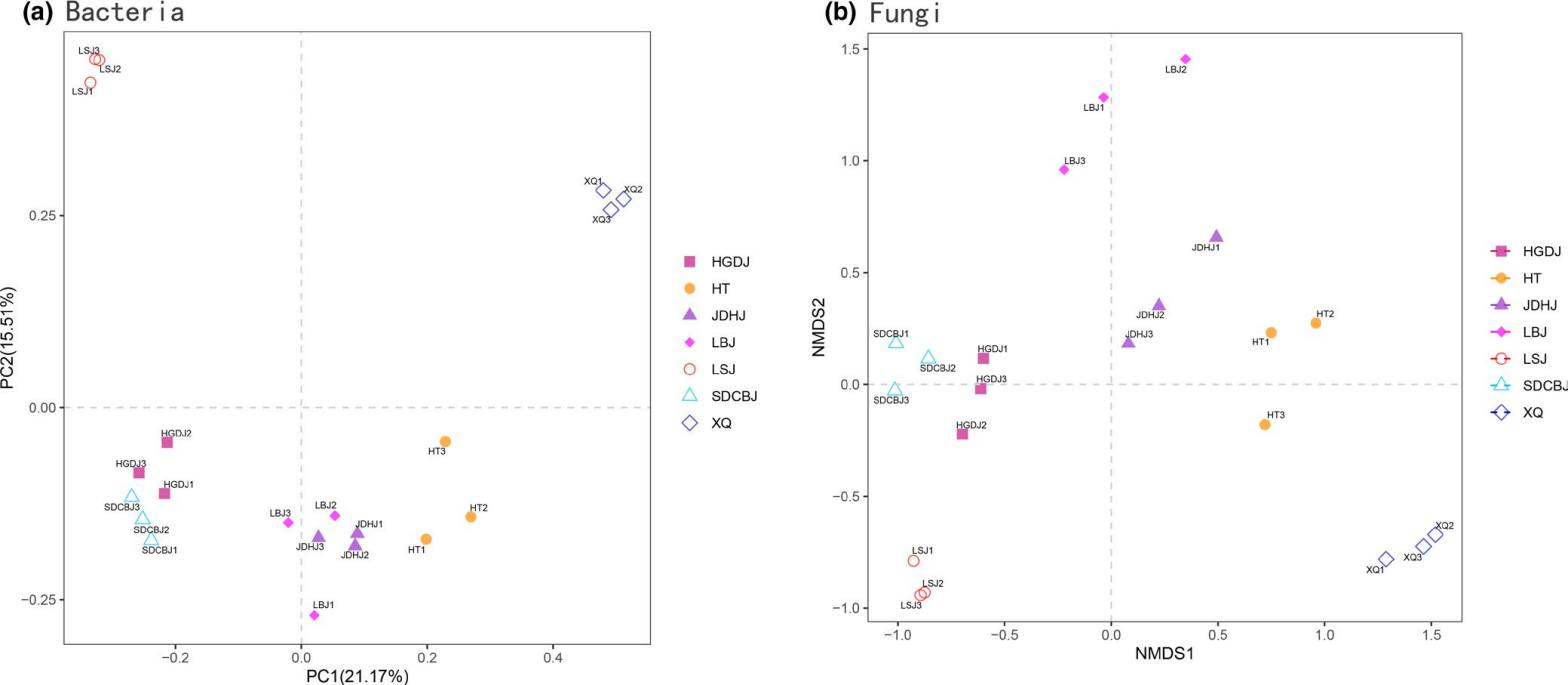
β diversity of microbial communities of Doujiang samples: (a) PCoA based on the relative abundance of bacterial OTUs showing the bacterial community structure; (b) NMDS showing the fungal community structure of different samples

PCoA (principal coordinate analysis) showed that each kind of Doujiang bacterial community formed individual cluster (Figure 4a). NMDS (nonmetric multidimensional scaling analysis) also illustrated the fungal community of different samples formed different clusters (Figure 4b). PERMANOVA also verified the significant differences in community compositions among the Doujiang samples (*p* < .05 in all pairs).

**FIGURE 5:**
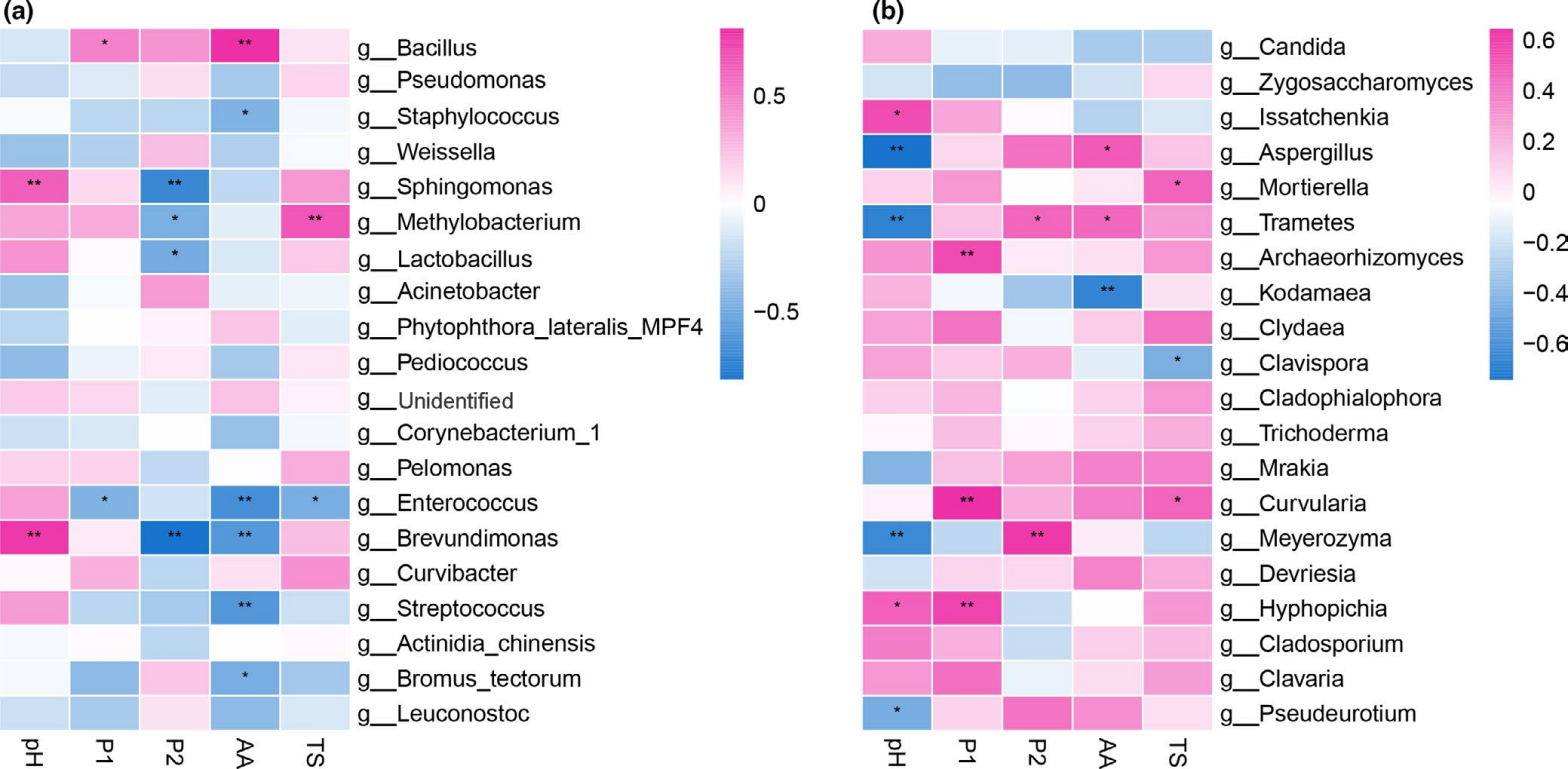
Spearman correlation heatmap: P1—total protein, P2—Soluble protein, AA—amino acid, TS—total sugar. *0.01 < *p* ≤ .05,**0.001 < *p* ≤ .01

The Spearman correlation heatmap illustrated that the microbial community structure (Top 20 genera) was remarkably correlated with Doujiang pH, content of total protein (P1), soluble protein (P2), amino acid (AA), and total sugar (TS) (*0.01 < *p* ≤ .05,**0.001 < *p* ≤ .01) (Figure 5). *Bacillus*, *Enterococcus*, *Lactobacillus,* etc., were remarkably related to the protein contents of Doujiang. The properties of Doujiang samples were listed in Table S2.

### 3.4 Isolated strains and protease activity of microbial strains

Fifty‐three bacterial and thirteen fungal strains were isolated from Doujiang samples (Table 2). Bacterial genera belong to *Bacillus* most, strains belong to *Lactobacillus* and *Oceanobacillus* were also cultured. Fungal genera belong to *Monascus, Candida,* and *Aspergillus*. Number in parentheses indicated duplicated isolated strains.

**TABLE 2:** Isolated strain of Doujiang samples

Sample number	Identification	Percent of Identity
Bacteria		
DJ1 (23)	*Bacillus subtilis*	100%
DJ24 (6)	*Bacillus velezensis*	99%
DJ30 (16)	*Bacillus amyloliquefaciens*	99%
DJ46 (2)	*Oceanobacillus* sp.	99%
DJ48 (2)	*Bacillus licheniformis*	100%
DJ50 (2)	*Bacillus siamensis*	100%
DJ52 (2)	*Lactobacillus plantarum*	99%
Fungi		
DJ54 (5)	*Monascus purpureus*	99%
DJ59 (5)	*Candida utilis*	99%
DJ64 (3)	*Aspergillus* sp.	99%

**FIGURE 6:**
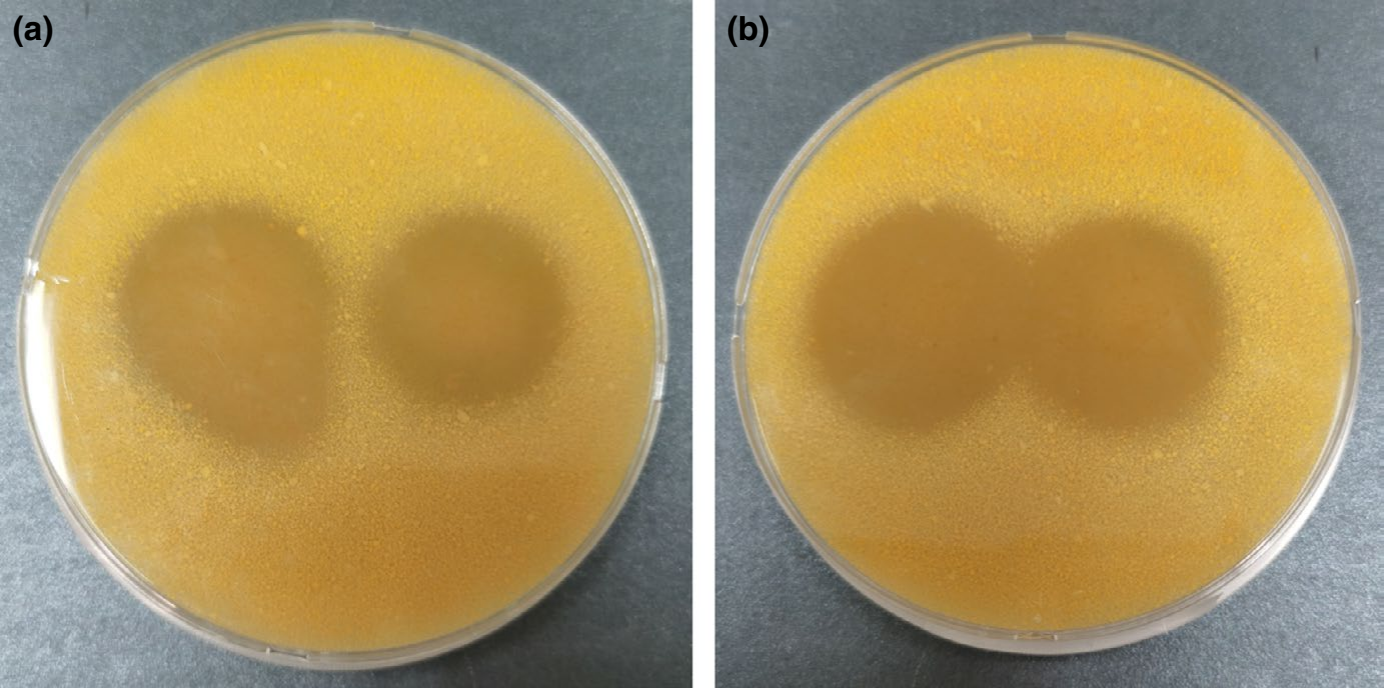
Hydrolytic transparent circles of some strains of corn gluten meal screening medium. (a) Left: DJ1, right: DJ5; (b) left:DJ8, right: DJ15

The strains were screened by corn gluten meal medium and Folinol method. Eleven strains showed transparent hydrolytic circles from corn gluten meal medium plates (Some were shown in Figure 6) and showed high protease activity (Table 3).

**TABLE 3:** Protease activity of microbial strains (U/ml)

Strain Codes	Acid protease activity	Neutral protease activity	Alkaline protease activity
DJ1	58.2	161.4	89.4
DJ3	59.9	97.9	101.7
DJ5	57.2	86.7	95.7
DJ7	64.9	95.7	104.4
DJ8	48.3	130.8	123.9
DJ12	41.7	72.1	58.2
DJ15	63.8	89.3	89.7
DJ24	53.4	102.1	108.2
DJ46	39.9	83.4	78.4
DJ48	42.7	125.9	72.2
DJ50	39.8	83.4	78.4

## 4 DISCUSSION

Traditional fermented soybean paste is very important in the dietary of many Asian countries (Gao et al., 2013; Kim et al., 2009; Zhang et al., 2018). Due to the special flavor and rich nutrition, people liked Doujiang very much (Gao et al., 2013; Kim et al., 2009; Zhang et al., 2018). Many previous studies of soybean paste have paid more attention to production process, sterilization technology, flavor quality, volatile components, etc. (Huang et al., 2010; Zhang et al.,^2018^, ^2019^). Recently, more and more concerns were given to microflora of soy paste with the realization of microbe importance and the development of biotechnology (Gao et al., 2013; He et al., 2017; Kim et al., 2009; Zhang et al.,^2018^, ^2019^), but these studies are still very limited to understanding microbial structure and functions in Doujiang. Much more researches and information are needed for further study and utilization. In our study, microflora of seven kinds of Doujiang from different regions were studied with both Illumina sequencing and culture‐dependent method. A total of 1,092,983 bacterial and 1,417,409 fungal sequences were generated, classified into 17 phyla and 681 OTUs (bacteria) and 7 phyla and 1,149 OTUs (fungi), illustrating an integrated picture for the few explored microbial diversity of traditional fermented soybean paste (Doujiang). The investigation will provide valuable references for other studies on microflora of soybean paste as well.

Bacterial Firmicutes and Proteobacteria were most abundant in our study, and the fungal communities were dominated by Ascomycota. The result was consistent with some previous studies of microbial diversity of fermented food, that is, soy sauce (Ma et al., 2019). Proteobacteria, Firmicutes, and Ascomycota were widespread; they are dominant microbial group in many other environmental systems such as plants and soils (Costa et al., 2021; Delgado‐Baquerizo et al., 2018; Muller et al., 2016). This may be due to the broad species diversity, adaptable capability, and faster evolutionary rate of the taxa (Wang et al., 2010). Class Bacilli, Gammaproteobacteria, and Alphaproteobacteria existed in highest abundance among the Doujiang samples. They were also common and ubiquitous bacteria groups widely distributed in many other environments (Geiser et al., 2015; Higgins et al., 2007; Maharachchikumbura et al., 2015).

Our study found that major genera were from *Bacillus* in prokaryotes, *Candida,* and *Aspergillus* in Eukaryotes, which were often composed of core OTUs in Doujiang microbiota. Genera *Bacillus*, *Lactobacillus*, and *Enterococcus* could be significant bacterial biomarkers for microbial community (LSJ) according to LEfSe analysis. While, abundance of *Aspergillus* (JDHJ), abundance of genus *Candida* in LSJ contributed most to their fungal structure differences. The high abundance might also indicate their active role in these samples. *Bacillus* is a group of rod‐shaped, gram‐positive, usually aerobic bacteria and can form dormant spores under adverse environment (Nakagawa et al., 2003). *Bacillus* are currently used as probiotic organisms (Duc et al., 2004; Schultz et al., 2017; Vinothkanna et al., 2021). *Bacillus* spp. existed in many fermented foods such as spices, cereals, soy sauce, and dried foods (Hong et al., 2008; Ma et al., 2019). *Candida* members including *C. utilis* are widely used in food fermentation (Yasuyuki et al., 2012). It has been engineered to confer a new biosynthetic pathway for producing carotenoids like lycopene, beta‐carotene, and astaxanthin (Miura et al., 1998). *Aspergillus* is very common in plants and soils, could be found on oranges and other fruits, and also be used in vinegar and vine production (*A. oryzae*) (Earl et al., 1981; Hong et al., 2004). *Lactobacillus* belongs to lactic acid bacteria (LAB) and is essential in food production (Ma et al., 2019; Zhao et al., 2009). *Lactobacillus* and their metabolites have been used to control foodborne pathogens and spoilage organisms in meat products ensuring food safety and quality (Swetwiwathana & Visessanguan, 2015), also as biopreservatives for foods, and improving postharvest quality of grapes (Fang et al., 2020). *Enterococcus* is Gram‐positive, nonspore‐forming, facultative anaerobic bacteria, inhabiting the alimentary tract of humans as well as environmental and animals (Fisher & Phillips, 2009). They could survive under a large range of stresses and hard environments (Fisher & Phillips, 2009). Enterocin produced by *Enterococcus* spp. has attracted great research interest due to the prevention function of pathogens (Cowled et al., 2020; Line et al., 2008).

Different soy bean paste samples might host different microbial communities (Zhao et al., 2009). Our results verify these findings to some extent, since each Doujiang sample may form a unique microenvironment and have different starter microbes (Gao et al., 2011; Ma et al., 2019; Zhao et al., 2009). For example, PCR‐DGGE microbial analysis of soybean paste from Anhui showed that *Bacillus megaterium*, *Lactobacillus plantarum*, *L. fermentum*, and *B. amyloliquefaciens* were predominant during fermentation (Zhao et al., 2009). Gao et al. (2013) found that bacteria of soybean paste were most related to *Leuconostoc* spp., *Lactococcus lactis*, *B. licheniformis*, and *Citrobacter freundii*. while *B. licheniformis* and *Leuconostoc* spp. were dominant during soybean paste fermentation of Tianyuan Jiangyuan (Gao et al., 2011). The main bacteria of soybean paste were *Staphylococcus*, *Enterobacter*, *Leuconosto*, *Bacillus, Chromohalobacter,* and *Lactobacillus*, and the main fungi wer*e Zygosaccharomycse*, *Aspergillus*, *Gibberella*, *Mucor,* and *Penicillium* (Ma et al., 2019). In our study, LSJ had the highest bacteria Chao 1 and Shannon index, while LBJ had the highest fungal richness Chao 1 and Shannon index, also showing their unique microbial community structure from another view. Traditional fermented food is great treasury for microbial community. Soybean paste also generates a complicated microbial community of fungi and bacteria during fermentation. Researches concerning microbial community composition and diversity of fermented food, especially various soybean paste should be enhanced constantly for future.

Microbial community composition was significantly correlated with Doujiang properties (Doujiang pH, content of total protein, soluble protein, amino acid, and total sugar). *Bacillus, Enterococcus, Lactobacillus,* etc., were remarkably related to Doujiang protein contents. Studies have found some microbes’ characteristic of the potential to produce vital aroma compounds, etc., showing their important role in fermentation and flavor (Gao et al., 2011; He et al., 2017; Zhao et al., 2009). For example, *Bacillus* can decompose proteins and starches of the raw materials with help of fungi, and *Leuconostoc* contribute to the compounds of flavor formation in soybean paste (Gao et al., 2011). Yeast members, for example, *Candida etchellsii* and *Kluyveromyces lactis*, could enhance flavors of alcohols, esters, and HEMF [4‐hydroxy‐2(or 5)‐ethyl‐5(or 2)‐methyl‐3(2H)‐furanone], etc. (Nakamura, 1990). These genera members also existed in our research. Their functions with Doujiang and relations with Doujiang properties merits further study. Moreover, Doujiang properties might also put on an effect on the microbial community composition. By the studies, a comprehensive view of the relationship between complicated microbial communities and the quality of products can be obtained (He et al., 2017). The producing area or geographic location could influence plant and food quality and flavor properties through their distinctive soil, water, and sunshine, etc. (Costa et al., 2021; Huang et al., 2010). Microbial community and flavor had a certain relationship with geographical location (Ma et al., 2019). Study has reported that the microbial community of Shandong soybean paste is different from soybean paste from Northeast (Gao et al., 2013; Ma et al., 2019). The producing area of seven kind of Doujiang in our study is from six regions (two kinds of samples are from same area), but the microbial community of each is distinctive. Moreover, the raw materials of soybean, including soybean cultivars and their genetics, might have influence as well. Thus, a great deal of more samples is needed to investigate to draw a more general conclusion.

As to culture‐dependent isolation, *Bacillus* spp. were most frequent ones. Genera *Lactobacillus and Oceanobacillus* (bacteria), *Monascus*, *Candida,* and *Aspergillus* (fungi) were also cultivated. Their existence can be verified in Illumina sequencing. Isolated strains are very limited with traditional method in this research. Traditional isolation could underestimate the complication of microbial ecosystems due to bias in selecting the microorganisms (He et al., 2017). It could also explain the results that our isolated strains are very limited compared to Illumina sequencing results, especially for fungi, to some degree. The high salt content and acid environment of Doujiang may be another reason. The presence of *Bacillus* spp., *Oceanobacillus* spp., and *Paenibacillus glycanilyticus* in the soybean samples may be due to their salt tolerance (Wu et al., 2013). High‐throughput sequencing (HTS) technology was a revolutionary innovation of gene sequencing and had broadened the scope of microbial analysis of environmental samples to a great extent (He et al., 2017; Mardis, 2008). Although a lot of attentions have been given to employing the techniques in food fermentation systems to investigate the microbial composition, dynamics, and to discover novel low‐abundant taxonomic lineages (Gao et al., 2013; He et al., 2017; Rizo et al., 2018). The structure and diversity of microbial community in the soybean paste should be deeply and broadly investigated continuously. At the same time, to isolate and get the strains is very important as well since the strains are the base for utilization (Menezes et al., 2020; Nahidul‐Islam et al., 2018). Corn gluten meal is main byproduct of corn wet‐milling process in the starch industry and contains 62%‐71% protein (Li et al., 2019; Zhu et al., 2019). Microbial fermentation to produce bioactive peptides with antioxidant, antihypertensive, hepatoprotective, anticancer, etc., activities is a useful and effective way to use the byproduct (Li et al., 2019; Zhu et al., 2019). We have been screening the isolated strains by corn gluten meal medium, and many strains showed transparent hydrolytic circles from corn gluten meal medium plates and had high protease activity, showing great potential to comprehensive utilization of corn gluten meal. The research could provide theoretical understructure for future production, quality improvement, and biopreservation of soybean paste, and the breeding of excellent strains as well.

## CONFLICT OF INTEREST

The authors declare no conflict of interest.

## ETHICAL STATEMENT

The study does not involve animal or human subjects.

## Supporting information

Fig S1

Fig S2

Table S1

Table S2

Supplementary Material

## Data Availability

The data that support the findings of high‐throughput sequences were submitted to the SRA of the NCBI under project accession number PRJNA688022. Sequences of cultured strains were under NCBI no. MW368498‐MW368550 and MW368653‐MW368665.
