# Supplementary figures and images for: Bacterial and fungal communities of traditional fermented Chinese soybean paste (Doujiang) and their properties
Source: Food Sci Nutr. 2021 Aug 31;9(10):5457–66. doi: 10.1002/fsn3.2505 (PMC8498056; doi:10.1002/fsn3.2505)

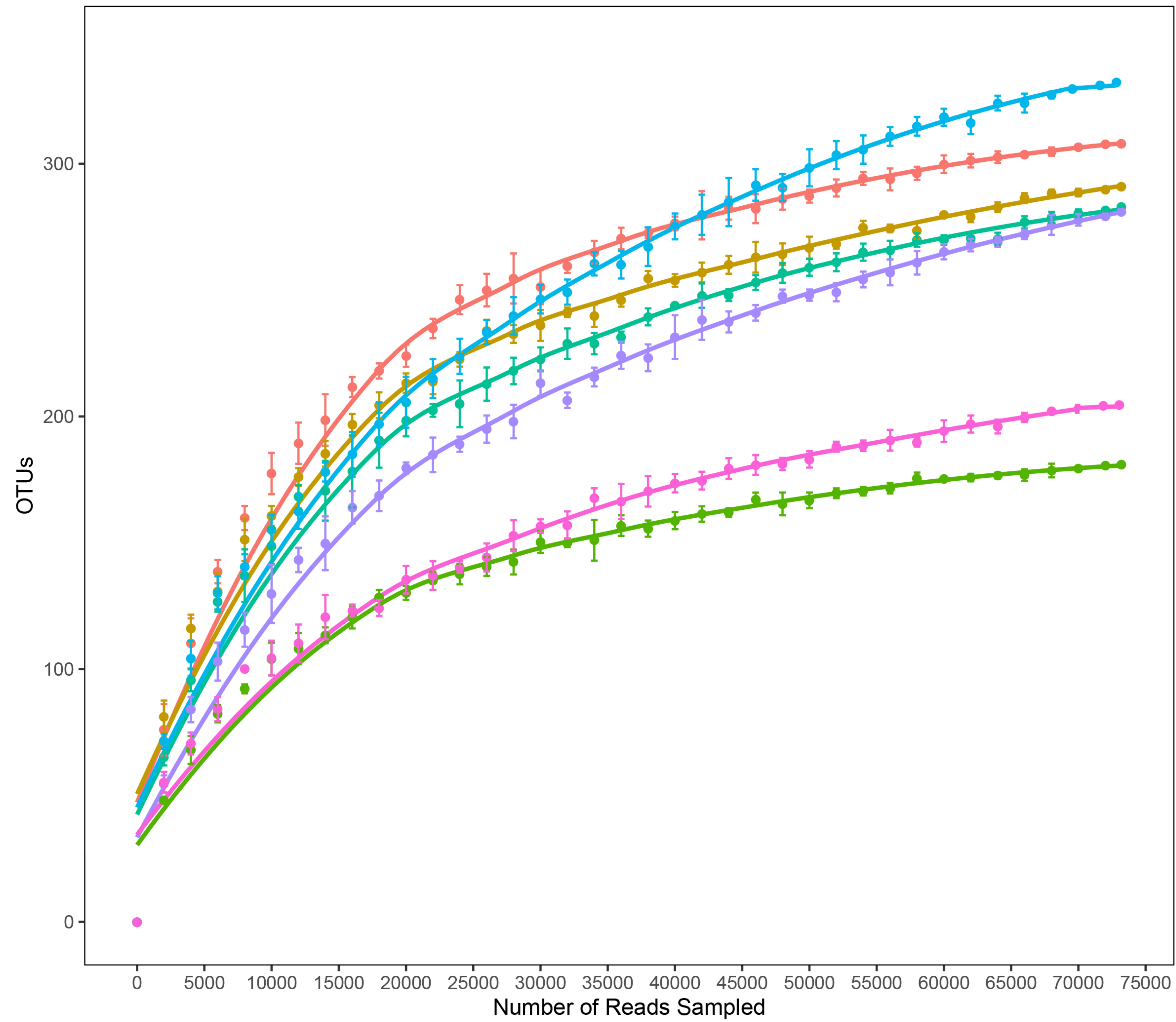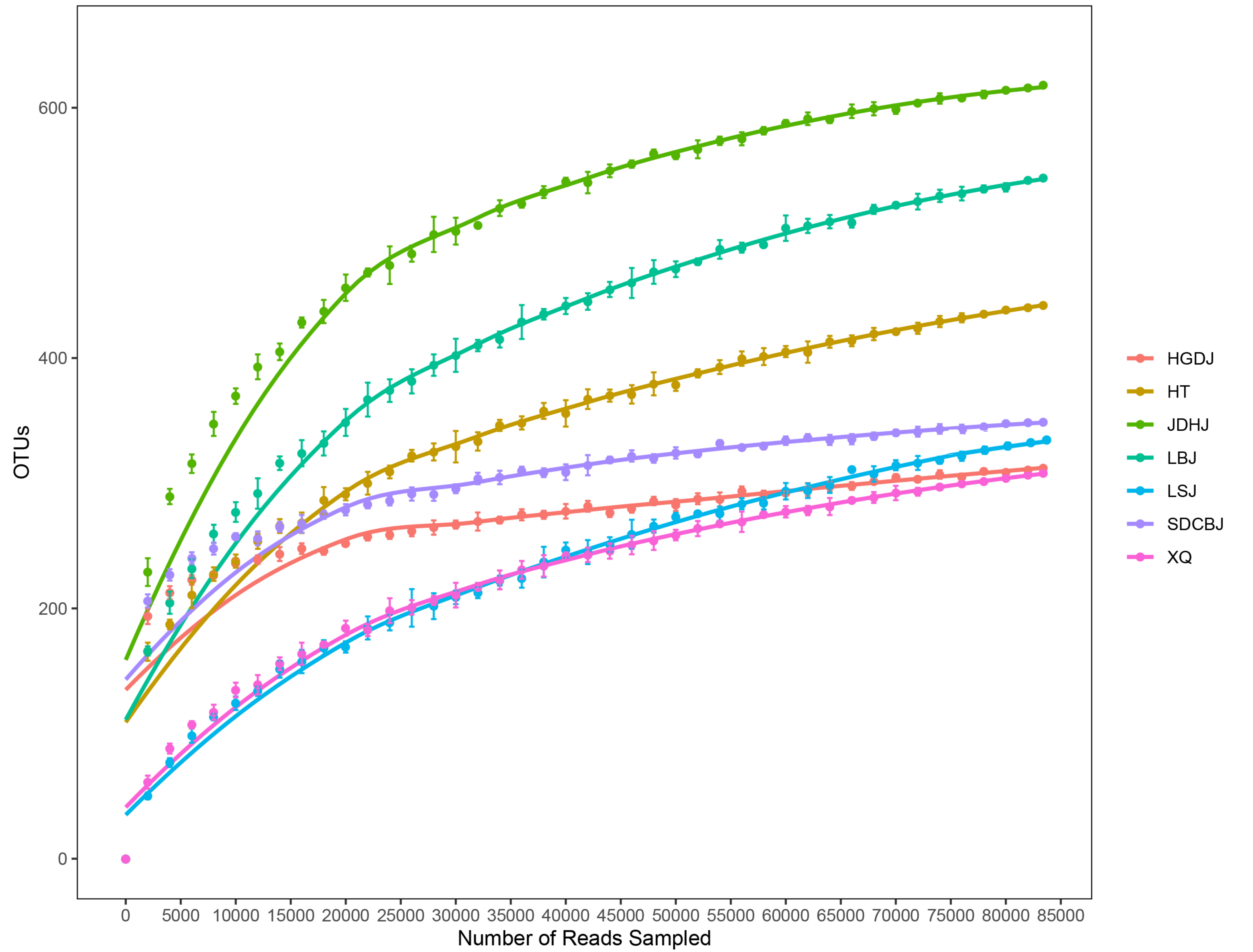

Supplement: Supplementary file 1 — Fig S1 [file FSN3-9-5457-s003.pdf]
