# Supplementary material for: Bacterial and fungal communities of traditional fermented Chinese soybean paste (Doujiang) and their properties
Source: Food Sci Nutr. 2021 Aug 31;9(10):5457–66. doi: 10.1002/fsn3.2505 (PMC8498056; doi:10.1002/fsn3.2505)
Supplement: Supplementary file 3 — Table S1 [file FSN3-9-5457-s001.docx]

**Supplementary Table 1 Doujiang samples resource information**

| Samples | Resource provinces |
| --- | --- |
| JDHJ | Beijing |
| LBJ | Beijing |
| XQ | Heilongjiang |
| LSJ | Hebei |
| HT | Guangdong |
| SDCBJ | Shandong |
| HGDJ | Jilin |
