# Supplementary material for: Bacterial and fungal communities of traditional fermented Chinese soybean paste (Doujiang) and their properties
Source: Food Sci Nutr. 2021 Aug 31;9(10):5457–66. doi: 10.1002/fsn3.2505 (PMC8498056; doi:10.1002/fsn3.2505)
Supplement: Supplementary file 4 — Table S2 [file FSN3-9-5457-s002.docx]

**Supplementary Table 2 Properties of Doujiang samples**

| Samples | pH | P1 (mg/g) | P2 (mg/g) | AA (μg/g) | TS(mg/g) |
| --- | --- | --- | --- | --- | --- |
| JDHJ | 4.01±0.02 | 3.363±0.003 | 0.6655±0.0002 | 1126.24±0.06 | 169.374±0.003 |
| LBJ | 4.08±0.01 | 2.962±0.002 | 0.5695±0.0002 | 839.55±0.05 | 81.434±0.003 |
| XQ | 4.26±0.01 | 1.595±0.001 | 0.6202±0.0001 | 1017.21±0.08 | 82.098±0.002 |
| LSJ | 4.16±0.03 | 2.858±0.001 | 0.5445±0.0002 | 389.12±0.02 | 68.708±0.002 |
| HT | 4.48±0.01 | 1.128±0.002 | 0.5915±0.0002 | 491.37±0.01 | 164.708±0.003 |
| SDCBJ | 5.33±0.02 | 2.094±0.002 | 0.6211±0.0003 | 686.204±0.03 | 76.703±0.002 |
| HGDJ | 5.15±0.01 | 1.307±0.002 | 0.6275±0.0002 | 512.12±0.02 | 174.258±0.003 |

*P1— total protein, P2—Soluble protein, AA—amino acid, TS—total sugar.
