# Supplementary material for: Bacterial and fungal communities of traditional fermented Chinese soybean paste (Doujiang) and their properties
Source: Food Sci Nutr. 2021 Aug 31;9(10):5457–66. doi: 10.1002/fsn3.2505 (PMC8498056; doi:10.1002/fsn3.2505)
Supplement: Supplementary file 5 — Supplementary Material [file FSN3-9-5457-s004.docx]

**Supplementary Materials:**

**Figure S1:** Rarefaction curves of Doujiang samples.

**Figure S2:** LEfSe analysis showing the significant microorganisms at different taxonomic levels. Bacteria (A) Cladogram of different taxa, nodes of different color represent significantly enriched and important taxa among Doujiang samples, (B) LDA graph, the higher LDA scores of the taxa represent the more contribution, fungal (C) Cladogram of different taxa, (D) LDA graph. Table 1. Doujiang samples resource information. Table 2. Properties of Doujiang samples.
