# Supplementary material for: Bacterial and fungal communities of traditional fermented Chinese soybean paste (Doujiang) and their properties
Source: Food Sci Nutr. 2021 Aug 31;9(10):5457–66. doi: 10.1002/fsn3.2505 (PMC8498056; doi:10.1002/fsn3.2505)
Supplement: Supplementary file 2 — Fig S2 [file FSN3-9-5457-s005.pdf]

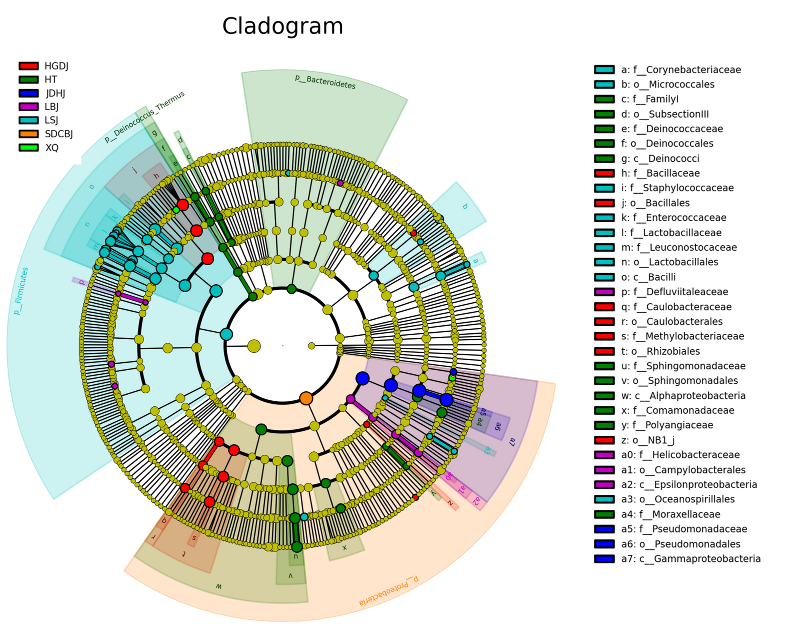
Cladogram

a

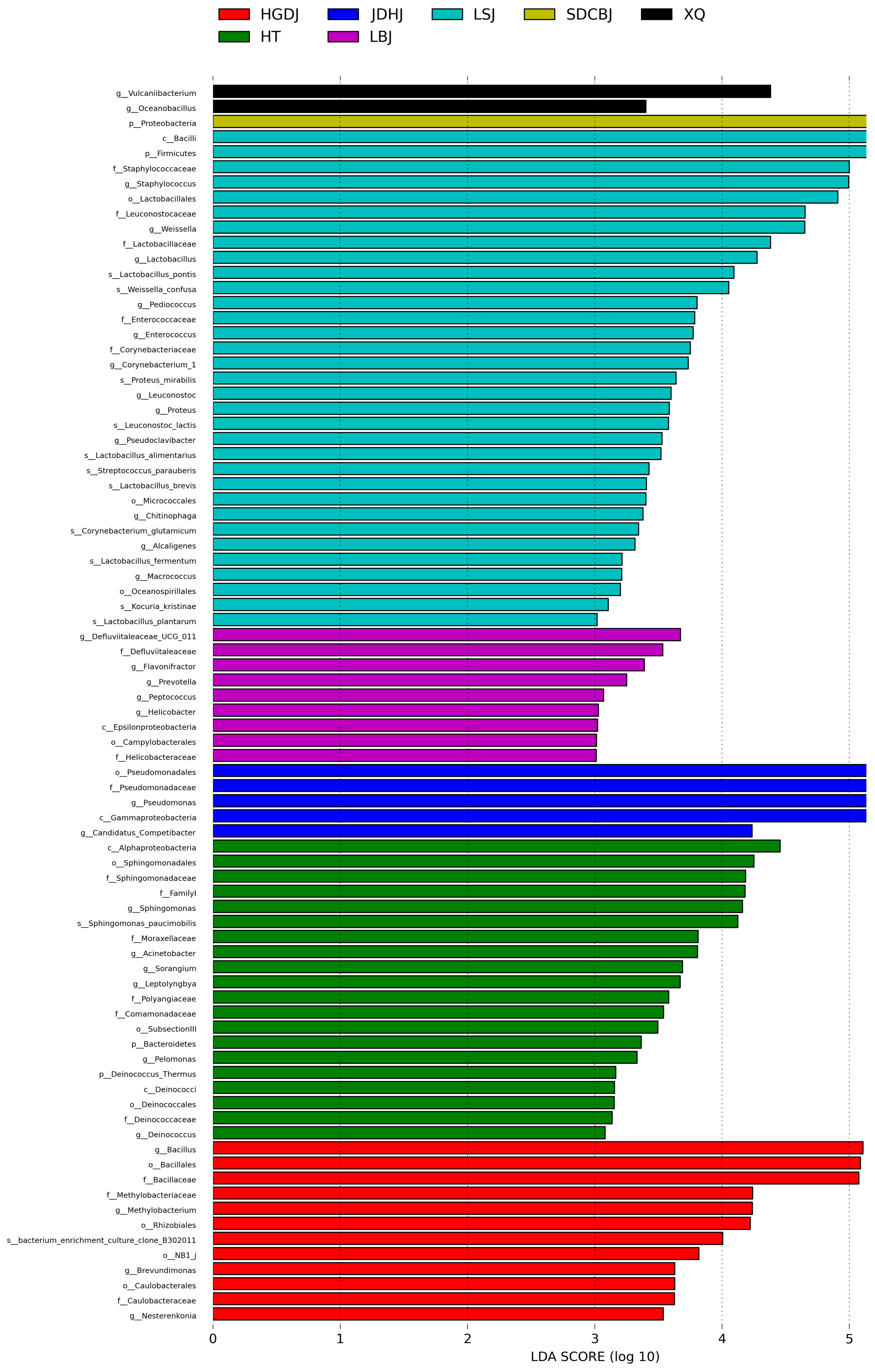
HGDJ
HT
JDHJ
LBJ
LSJ
SDCBJ
XQ
LDA SCORE (log 10)

b

Fungal

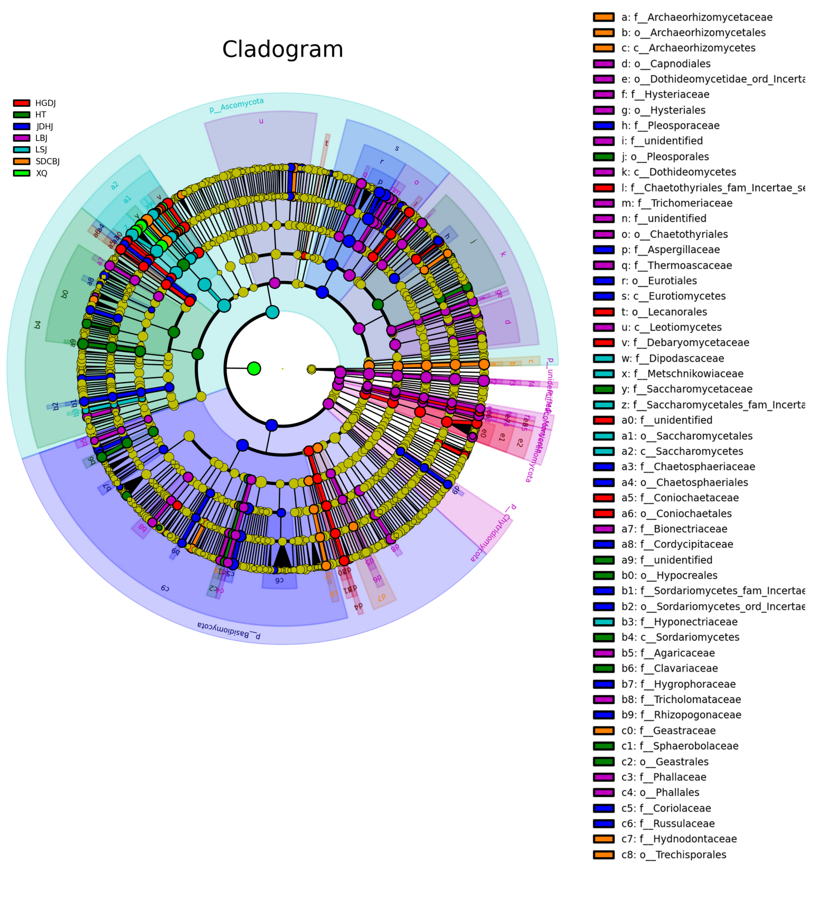
Cladogram

c

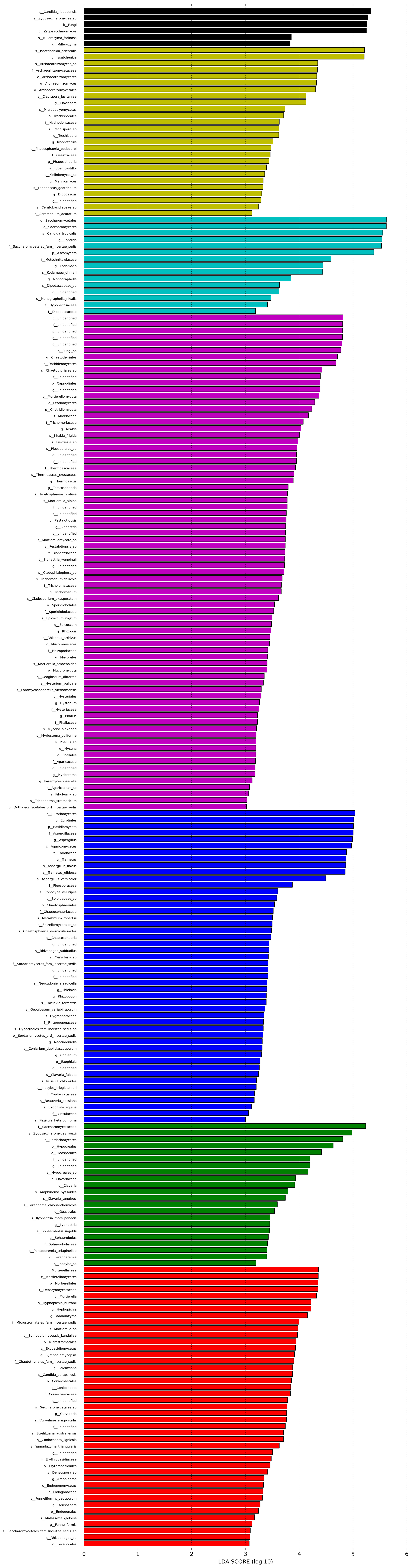
LDA SCORE (log 10)

d
